# The Effect of Hydrogen Peroxide With Different Concentration on the Color and Surface Microhardness of the Resin Bracket

**DOI:** 10.1002/cre2.70210

**Published:** 2025-09-08

**Authors:** Song‐Yi Yang

**Affiliations:** ^1^ Department of Dental Hygiene Konyang University Daejeon Republic of Korea

**Keywords:** hydrogen peroxide, microhardness, resin bracket, shear bond strength, whitening

## Abstract

**Objectives:**

This study aimed to evaluate the whitening effect, shear bond strength (SBS), microhardness, and microstructure of discolored resin brackets following whitening treatment with various concentrations of hydrogen peroxide (HP).

**Material and Methods:**

Resin brackets were bonded to the enamel surface and discolored with a curry solution. Control (distilled water; DW) and experimental solutions of 8.7%, 17.5%, and 35% HP were applied to the discolored resin brackets for 15 min. Resin brackets were then stored in DW for a week by repeating this whitening process three times. The whitening efficacy was evaluated according to the ISO 28399:2011. The SBS was measured using a universal testing machine. The microhardness and microstructure of the resin brackets were observed using a microhardness tester and field‐emission scanning electron microscopy (FE‐SEM). All the results were analyzed using a one‐way ANOVA analysis and Tukey's post‐hoc test (*p* = 0.05).

**Results:**

All the experimental groups demonstrated a significant whitening effect on the discolored resin brackets compared to the control group (*p* < 0.05). Especially, 35% HP showed faster color changes than the other groups (*p* < 0.05). The microhardness of the resin bracket was significantly lower in 35% HP compared to the control group (*p* < 0.05). FE‐SEM analysis revealed no significant difference between the experimental and control groups.

**Conclusions:**

HP application at concentrations of > 8.7% and < 17.5% is effective in whitening discolored resin brackets while not deteriorating the SBS, surface hardness, and microstructure. Dental clinicians can safely use HP within the recommended range to achieve effective esthetic outcomes without compromising bracket performance and durability.

## 1 Introduction

Many individuals with a desire for a harmonious and aligned smile seek orthodontic treatment to satisfy the esthetic preferences of a modern society (Gomes et al. 2017). Orthodontic appliances can be classified as removable and fixed orthodontic appliances, which are further classified into intraoral, interoral, and extraoral orthodontic appliances according to the site of the resistance source (Brandão et al. 2015). Brackets, which are fixed orthodontic appliances, are the most commonly used orthodontic treatment. Brackets vary based on the material, namely metal, resin, ceramic, and other composites. In particular, the use of ceramic or resin brackets, which are similar to the tooth color, is increasing to meet the esthetic demands of the patients.

Ceramic brackets are known for their superior resistance to discoloration and wear, and offer excellent esthetic properties. However, their strong adhesion to the tooth surface can lead to significant discomfort during removal and an increased risk of enamel damage (Kitahara‐Céia et al. 2008). Resin‐based brackets, on the other hand, offer comparable esthetics while achieving optimal bond strength, which facilitates removal and reduces the possibility of enamel damage (Faltermeier, Behr, and Müssig 2007a). However, resin‐based brackets remain susceptible to discoloration from dietary stains, highlighting the need for further research into this limitation.

Color changes in resin composites are affected by both exogenous and endogenous factors. Endogenous discoloration is related to many aspects of the composition of the bracket material, such as the resin matrix, filler particle content, and initiator/co‐initiator system (Schneider et al. 2011). Extrinsic factors for discoloration include staining by adsorption or absorption of colorants as a result of contamination from an exogenous source (Um and Ruyter 1991). The material, structure, filler content, and surface roughness play decisive roles in the extent of external discoloration (Lu et al. 2005). Extrinsic factors for discoloration are known to cause staining of the oral tissues and restorations, especially when combined with dietary factors. Among them, coffee, tea, nicotine, and curries have been reported (Um and Ruyter 1991; Guler et al. 2005). This is because the crystal lattice structure of the resin has high internal energy; therefore, a solution with a high concentration is absorbed by the resin during the absorption process (Wozniak et al. 1981). Therefore, the higher the concentration of dietary factors, the greater is the effect on resin discoloration (Al‐Samadani 2013). Absorbed dietary factors have been attributed to the continued formation of colored reaction products. In addition, abrasion or chemical erosion can occur, which increases the possibility of foreign matter staining on the surface (Powers et al. 1985).

The low color stability of resin brackets does not satisfy the esthetic needs of the orthodontic patients. This creates an opportunity for orthodontic patients to increase their concerns regarding bracket surface (Jadad et al. 2011; Çörekçi et al. 2010). Many studies have demonstrated the effectiveness of whitening agents on teeth and their effect on the bond strength with brackets. However, studies on the effect of whitening agents on discolored resin brackets, and color change, bonding strength with the enamel, and surface of the resin brackets are lacking.

Therefore, this study aimed to investigate the whitening effect of varying concentrations of hydrogen peroxide (HP), a major component of bleaching agents, on resin brackets and comprehensively evaluate the bond strength of enamel and resin brackets as well as the microhardness and microstructural aspects of the bracket surface following whitening treatment.

The null hypothesis was that the concentration of HP does not affect the color, shear bond strength (SBS) with the enamel, and microhardness and microstructural aspects of the discolored resin bracket surface.

## 2 Materials and Methods

### 2.1 Specimen Preparation

Forty extracted bovine incisors were selected for this study. These teeth were obtained from a commercial supplier of biological resources, TSS, based in Incheon, Korea, and were not obtained directly by the authors. All teeth were provided in accordance with the established procedures of the supplier. The selection criteria included teeth with sound enamel and the absence of fractures or caries. The roots of the bovine teeth were cut to leave only the crown, and the organic and foreign matter remaining on the surface was removed using a diamond cutter. The bovine crown was embedded in acrylic resin, and the labial side was exposed. After setting the acrylic resin, the labial surface was ground using a polishing machine (EcoMet 30 Manual Single Grinder Polisher, Buehler, Illinois, USA) with No. 1200 silicon carbide abrasive paper to obtain a uniform parallel enamel surface. All the specimens were polished to minimize the effect of the surface conditions on the measured values.

### 2.2 Resin Bracket Bonding to Enamel Surface

The exposed enamel surface was etched with 37% phosphoric acid (DentiAnn Etch Plus 37% Phosphoric Acid Etchant, Seil Global Co., LTD, Busan, Korea) for 30 s. It was then rinsed with a 3 way‐syringe for 30 s and dried for 10 s until the etched enamel exhibited a chalky white appearance. Transbond XT primer (3 M Unitek, Monrovia, CA, USA) was applied to the etched enamel surface, and a gentle flow of air for 2 s was subsequently applied to the etched enamel surface. It was then light‐cured for 10 s using a light‐curing unit (DCL‐30, DENTALL Co. Ltd., Bucheon, Korea). Transbond XT adhesive (3 M Unitek, Monrovia, CA, USA) was applied to the resin bracket base (Shine M Clear Resin Standard Bracket 022, Orthodontic DAESEUNG Medical, Seoul, Korea) and placed at the center of the enamel surface. To standardize the bonding adhesive thickness and pressure, a 1 pound Gilmore needle was held vertically on the bracket and the excess adhesive around the bracket was removed with an explorer before polymerization. Following the manufacturer's recommendations, the light‐curing unit was applied in four directions (gingival, incisal, distal, and mesial) for a minimum of 10 s per direction to ensure uniform and thorough polymerization across all bracket surfaces.

### 2.3 Staining Procedure

The color source was prepared by stirring 50 g of curry (Ottogi Curry Medium; Ottogi, Anyang, Korea) in 350 mL of distilled water for 30 min. Each enamel specimen attached to the resin bracket was entirely immersed in a well plate containing 4 mL of curry solution. The colored samples were stored in a constant temperature water bath (Water Bath WBA‐1, Chang Shin Scientific Co., Seoul, Korea) at 37°C for 14 days to replicate an environment similar to the oral environment. The coloring source was replaced daily with fresh curry solution at a constant time. After staining, the tooth specimen attached to the resin bracket was removed from the coloring solution, washed with distilled water, and dried.

### 2.4 Whitening Procedure

Specimens that were colored with the curry solution were randomly selected and classified into four groups. The experimental whitening solution was prepared by diluting distilled water with 35% HP (Seongwang Hydrogen Peroxide; Firson Co. Ltd., Cheonan, Korea) (Table 1). This study was designed with three experimental groups of 8.7%, 17.5%, 35%, and one control group of distilled water (HP 0). The stained specimens, consisting of resin brackets bonded to enamel, were immersed in the experimental whitening and control solutions, ensuring that both the brackets and the surrounding enamel were exposed to the HP treatment. The specimens were then stored in a constant‐temperature water bath at 37°C for 15 min per application. Thus, the whitening treatment was performed three times at 1‐week intervals. The samples were placed in fresh distilled water and stored in a constant temperature water bath at 37°C until the subsequent application.

**Table 1:** The composition ratio of the experimental whitening solution (vol.%).

Group	35% H₂O₂ solution	Distilled water
HP 0	0	100
HP 8.7	25	75
HP 17.5	50	50
HP 35	100	0

### 2.5 Color Assessment

This study used visual observation to record the color changes in the brackets according to ISO 28399:2021. Color changes were recorded before and after each whitening treatment using a shade guide (VITA classical shade guide, VITA Zahnfabrik H. Rauter GmbH & Co., Bad Säckingen, Germany). The degree of contrast before and after whitening according to the number of application of the experimental and control solutions was recorded for each experimental group, according to the order of “VITA Classic” as follows in decreasing order of brightness: B1, A1, B2, D2, A2, C1, C2, D4, A3, D3, B3, A3.5, B4, C3, A4, and C4. The evaluations were performed under standardized conditions at a consistent site using an identical light source, with specimens positioned uniformly. All color changes were assessed by a single investigator who was trained and calibrated before the study to ensure consistency and accuracy. A whitening effect was considered when the shade appeared brighter by two or more shades compared with that before applying the experimental whitening and control solutions.

### 2.6 Shear Bond Strength

After the third whitening application, the experimental and control group samples were stored in distilled water at 37° C for 24 h, and the SBS was subsequently measured. The specimen was positioned parallel to the major axis of the measuring instrument, and a load was applied at a crosshead speed of 1 mm/min using a universal testing machine (Instron 5942, Instron, Massachusetts, USA) to measure the maximum load when the bracket was debonded. The maximum force required to debond the bracket was recorded in Newton (N) and converted into megapascals (MPa) by dividing the measured maximum load by the bracket surface area (9 mm^2^).

$$\text{SBS}\;(\text{MPa})=\frac{\mathrm{Maximum force}\;(N)}{\mathrm{Experimental bracket surface area}\;({\text{mm}}^{2})}$$

The enamel surface, with the brackets removed, was observed at 50× magnification using an image analyzer (KH‐1000, Hirox. Co. Ltd., Tokyo, Japan). The remaining adhesive remnants on the tooth surface were evaluated using the adhesive remnant index (ARI) described by Årtun and Bergland, and the fractured brackets were also observed (Årtun and Bergland 1984). ARI was scored as follows: 0, no adhesive left on the tooth; 1, < 1/2 of the adhesive left on the tooth; 2, > 1/2 of the adhesive left on the tooth; 3, all the adhesive left on the tooth, with a distinct impression of the bracket base.

### 2.7 Microhardness

After measuring the SBS, the microhardness of the separated bracket surface was determined using a microhardness tester (Digital Micro Vickers; MMT‐X7; Matsuzawa Co., Akita, Japan). The Vickers hardness number (VHN) was measured using an indentation formed by vertically applying a load of 100 g to the surface of the measurement point for a dwell time of 10 s using a diamond‐shaped indenter. The surface of each bracket was randomly measured four times to obtain representative values for each specimen.

### 2.8 Resin Bracket Surface Observation

After whitening, the resin bracket surface was sputter‐coated with platinum in a vacuum evaporator at 20 mA for 60 s and observed at 2000× and 10,000× magnification with an accelerating voltage of 10.0 kV using a field emission scanning electron microscope (FE‐SEM; JSM‐7800F; JEOL Ltd., Tokyo, Japan).

### 2.9 Statistical Analysis

Data were analyzed using the statistical analysis software SPSS (Version 28.0; SPSS Inc., Chicago, IL, USA). Before statistical analysis, normality and homogeneity of variance were assessed using the Shapiro‐Wilk and Levene's tests, respectively. As both assumptions were met (*p* > 0.05), one‐way analysis of variance (ANOVA) was performed to compare the results among groups based on the concentration of the experimental whitening agent. Tukey's post‐hoc test was applied for multiple comparisons. Statistical significance was set at α = 0.05.

## 3 Results

### 3.1 Color Assessment

**Figure 1:**
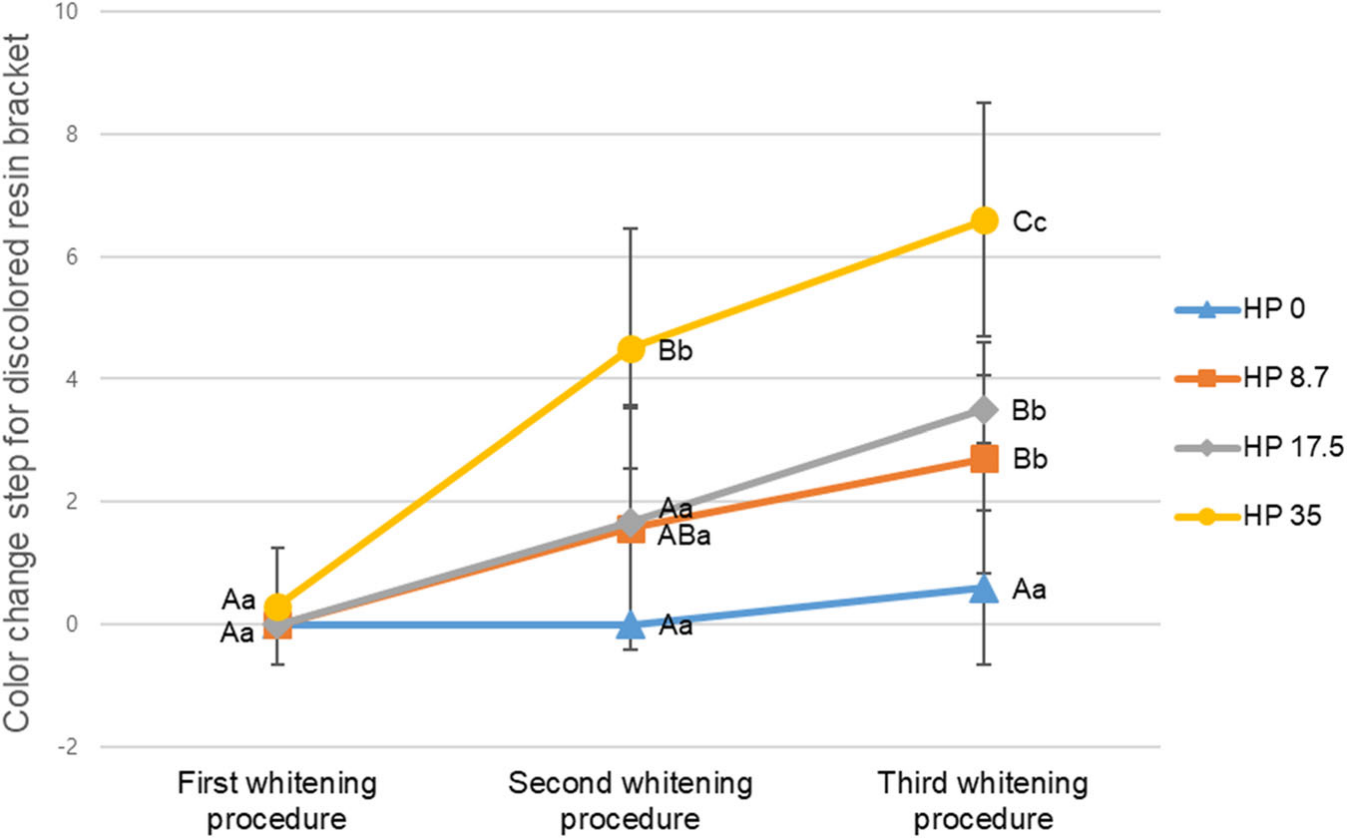
Steps of the color change of discolored resin brackets following application of the experimental and control solutions. Different lowercase letters indicate a significant difference in the color change between the whitening treatment procedures within the same experimental group; different capital letters indicate a significant difference in the color change between the different concentrations of HP within the same whitening treatment procedure.

**Figure 2:**
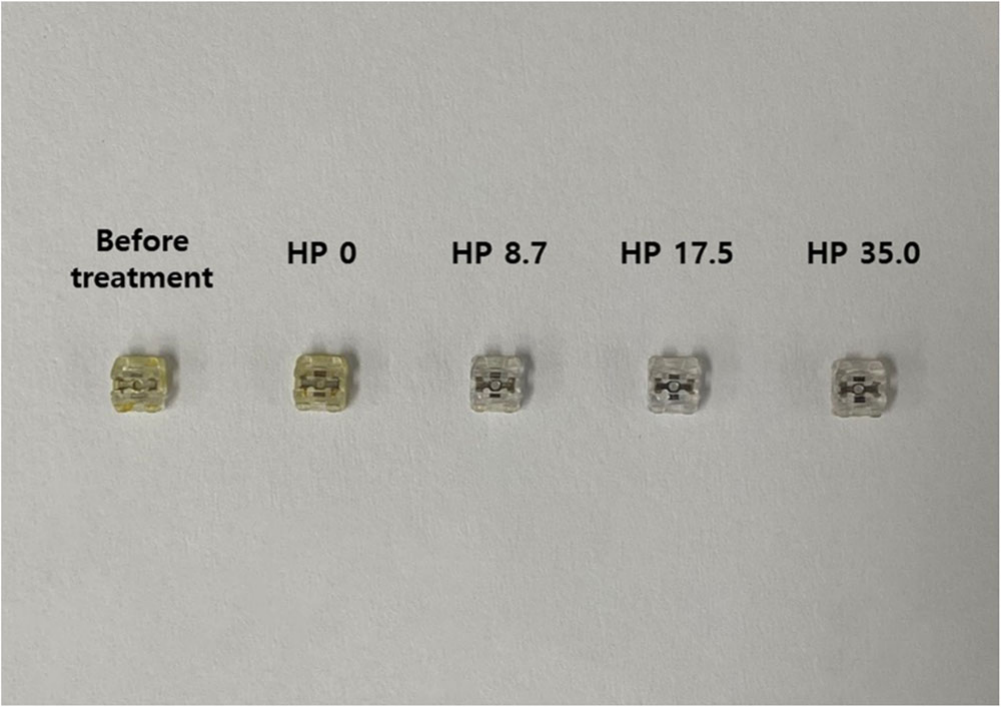
Final appearance of the discolored resin brackets following three whitening treatment applications with a control and experimental solution for 15 min.

Figure 1 shows the mean and standard deviation of the color change in the discolored resin brackets treated with the control and experimental solutions. Within the HP 0 group, no significant difference in the color change was observed at the whitening treatment stage (*p* > 0.05). Group HP 8.7 demonstrated a significant difference between the first and third whitening application procedure (*p* < 0.05). Group HP 17.5 demonstrated a significant difference between the first and third whitening application procedure and the second and third whitening application procedure (*p* < 0.05). Group HP 35 demonstrated a significant difference between the first, second, and third whitening application procedures (*p* < 0.05). In the first whitening treatment, no significant color change was observed at different concentrations of HP (*p* > 0.05). In the second whitening treatment application, Group HP 35 showed a significant difference from Groups HP 0, HP 8.7, and HP 17.5 (*p* < 0.05). The third whitening treatment application demonstrated a significant difference in all the groups except for the HP 8.7 and HP 17.5 groups (*p* < 0.05). In all the experimental groups, after the third whitening treatment, we observed an increase of more than two levels in the order of the shade guides by value, compared to before the whitening treatment (Figure 2). Therefore, all the experimental groups except the control group were found to demonstrate a whitening effect, especially HP 35, which showed a rapid whitening effect only after the second whitening treatment application.

### 3.2 Shear Bond Strength

**Figure 3:**
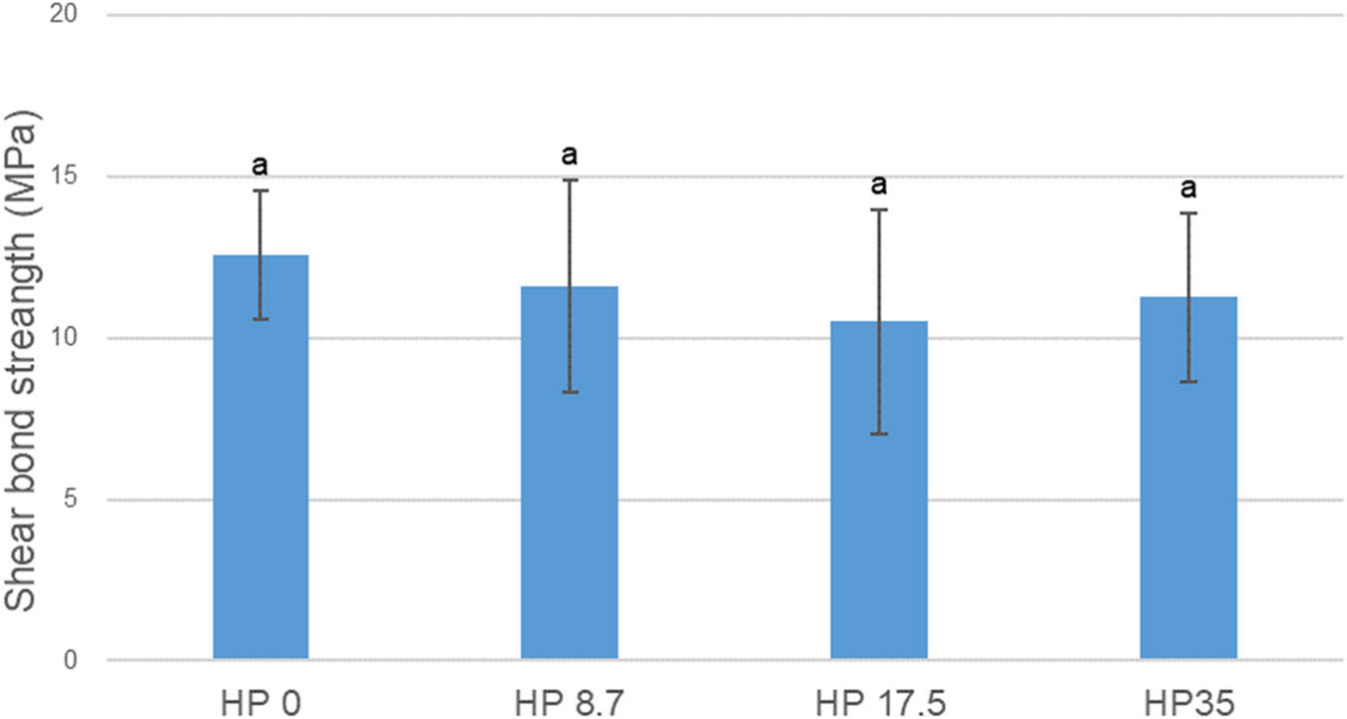
Shear bond strength between the resin bracket and enamel surface for each experimental and control groups (HP 0, HP 8.7, HP 17.5, and HP 35). The same lowercase letters indicate no significant difference between the experimental and control groups (*p* > 0.05).

**Figure 4:**
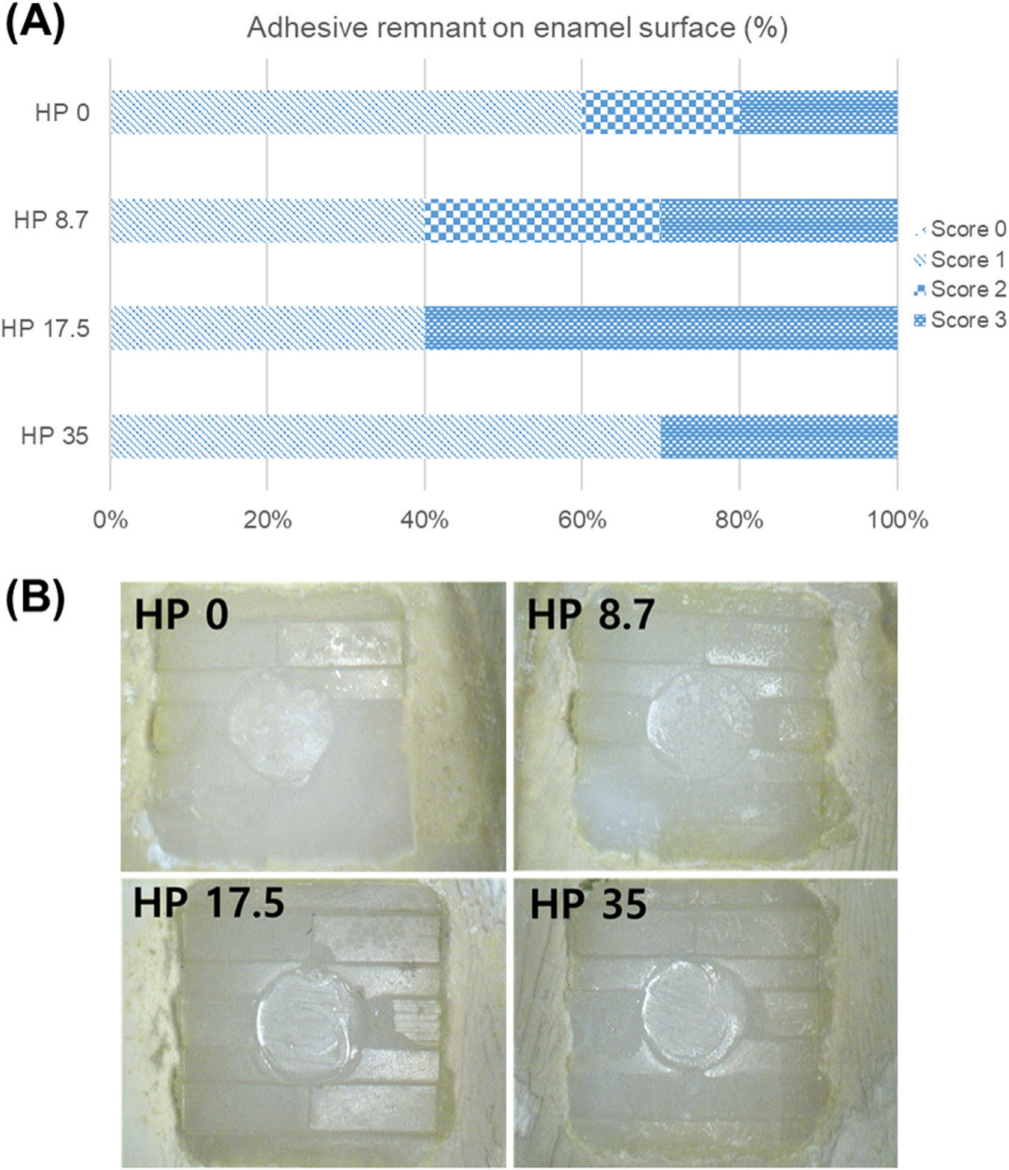
(A) Adhesive remnant score (% of total) for the debonded tooth surface. Adhesive remnant index (ARI) score 0: no adhesive left on the tooth, 1: < 1/2 of the adhesive left on the tooth, 2: > 1/2 of the adhesive left on the tooth, 3: all adhesive left on the tooth. (B) Enamel surface imaged by image analyzer following bracket removal.

The results of the SBS test according to the HP concentration are shown in Figure 3. The SBS of HP 0, HP 8.7, HP 17.5, and HP 35 were 12.57 ± 1.99 MPa, 11.58 ± 3.28 MPa, 10.50 ± 3.47 MPa, and 11.27 ± 2.61 MPa, respectively. No statistically significant differences between the experimental and control groups were observed (*p* > 0.05). Therefore, the HP concentration did not affect the SBS of the brackets or enamel. The ARI scores of the enamel surfaces are shown in Figure 4A. None of the groups scored 0. Groups HP 0, HP 8.7, and HP 35 demonstrated a higher frequency of score 1, which implies that less than 50% of the adhesive remained on the enamel surface. Group HP 17.5 had a greater frequency of a score of 3, which implies that all adhesive remained on the tooth, with a distinct impression of the bracket base (Figure 4B).

### 3.3 Microhardness of the Resin Bracket Surface

**Figure 5:**
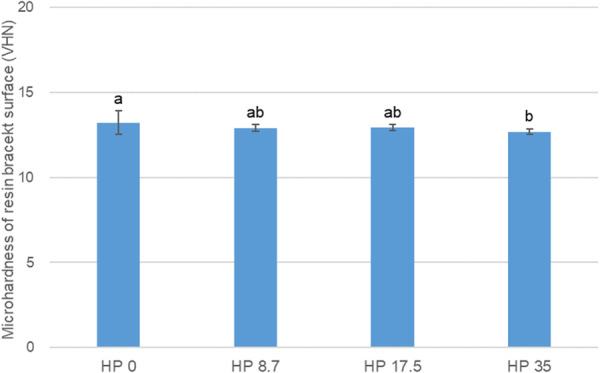
Surface microhardness (Vickers hardness number, VHN) of resin brackets following whitening treatment using control and experimental solution. The same lowercase letter indicates no significant differences in the VHN between the groups (*p* > 0.05).

The mean and standard deviation of the microhardness of the resin bracket surfaces for each group are shown in Figure 5. VHN of group HP 0 (13.23 ± 0.69) was the highest, and statistically significant differences were observed compared with group HP 35 (12.69 ± 0.17) (*p* < 0.05). However, no statistically significant difference was observed between group HP 8.7 (12.91 ± 0.21) and HP 17.5 (12.93 ± 0.17) (*p* > 0.05).

### 3.4 SEM Observations of the Resin Bracket Surfaces

**Figure 6:**
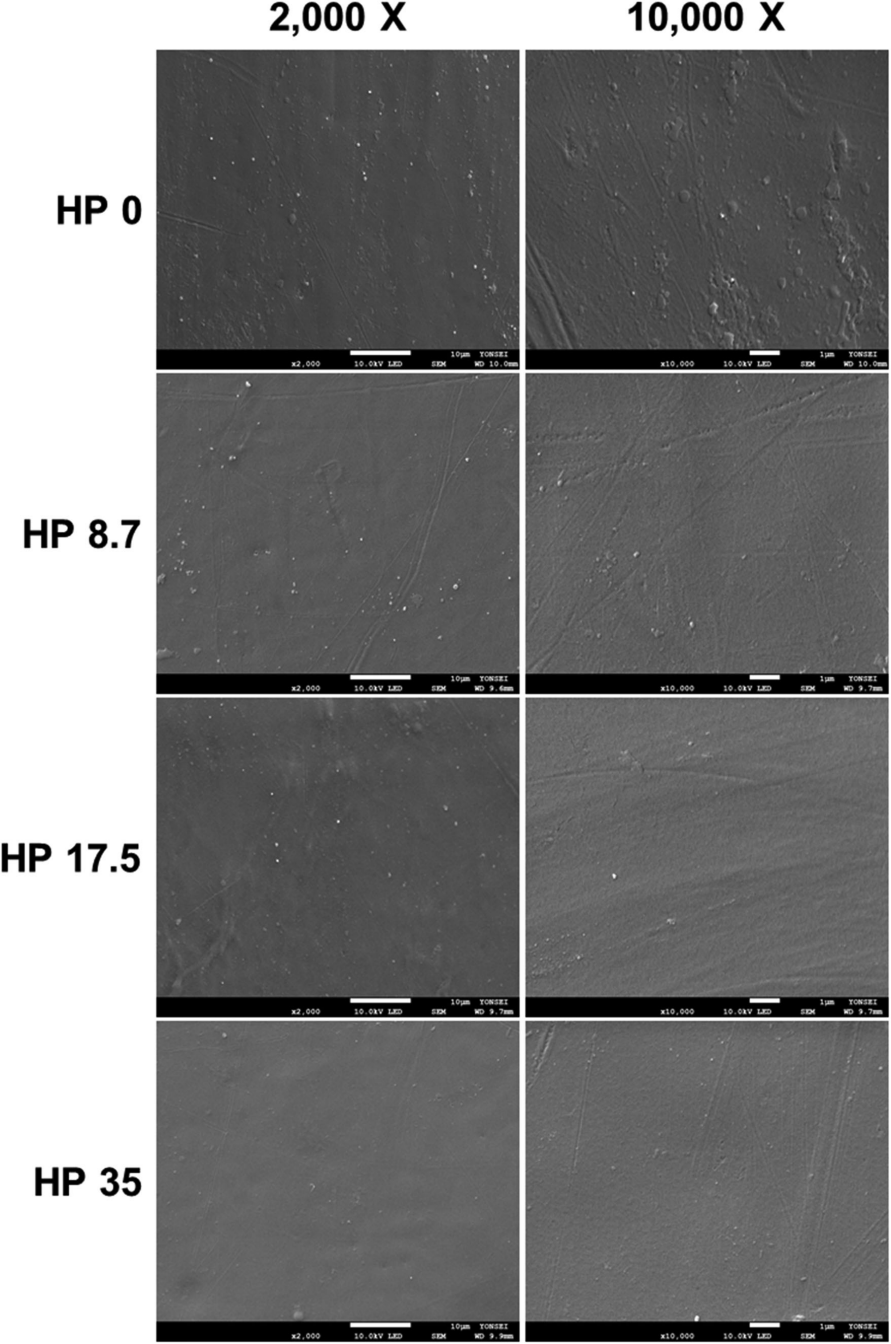
Representative scanning electron microscopy (SEM) images of each group following whitening treatment with different concentrations of hydrogen peroxide (HP) at 2000× (left) and 10000× (right).

SEM images of the resin bracket surfaces are shown in Figure 6. The resin brackets treated with the experimental and control solutions revealed no observable changes in their surface texture. The SEM images of the specimens exposed to the experimental solution revealed no resin bracket surface morphological alterations when compared to the SEM images of the control surfaces (HP 0).

## 4 Discussion

Incorrect brushing of brackets employed in orthodontic treatment could result in negative effects on the oral hygiene, creating a source of plaque accumulation and staining. In addition, the type of bracket, its composition, and tooth malposition can also affect maintenance of oral hygiene (Çörekçi et al. 2010; Balenseifen and Madonia 1970; Forsberg et al. 1991). Orthodontic brackets experience color stability challenges when exposed to staining substances for extended periods. In the case of resin brackets, which are widely used in orthodontic treatment owing to their esthetic appeal, their surface and physical properties can be affected by short‐term exposure to staining substances in the oral cavity due to their low abrasion resistance and insufficient fracture stability (Schulze et al. 2003; Eldiwany et al. 1995). This causes dissatisfaction and unmet esthetic demands of the patient, thereby resulting in the need for whitening treatment (Malkiewicz et al. 2022).

Among the previous studies related to whitening agents, the effect of whitening agents on tooth surfaces has been demonstrated in several studies. However, studies on the whitening effect, bond strength with teeth, and effect on the surface change of resin brackets upon application of whitening agents to discolored resin brackets owing to the surrounding oral environment are lacking (Alqahtani 2014; Abu‐Saq Al Yami et al. 2020; Ferreira et al. 2016). Therefore, this study aimed to investigate the whitening effect on the surface of resin brackets by applying different concentrations of HP, a major component of whitening agents, to discolored resin brackets, to comprehensively evaluate the SBS between the enamel and resin brackets, microhardness, and microstructural aspects of the bracket surface following whitening treatment, and determine the optimal concentration of HP for performing whitening in patients undergoing orthodontic treatment with resin brackets without compromising the surface properties of the brackets.

Discoloring materials are large pigment molecule composed of complex carbon double bonds that absorbs light in the visible light spectrum range (400–700 nm) to show color. Because HP, the main component of whitening agents, is unstable, it decomposes into water and active oxygen. These active oxygen species decompose and convert large pigmented molecules into small pigmented molecules via an oxidation reaction. The pigmented molecules converted in this manner do not absorb light in the visible light spectrum, resulting in a whitening effect (Basting et al. 2004).

Moreover, the dissolution products of HP are low‐molecular‐weight molecules that readily diffuse into the lamellae, grooves, fissures, and depressions present in the enamel structure, allowing the whitening agent to diffuse into the tubular structure of dentin, where the free radicals generated by HP act in multiple directions (Jadad et al. 2011; Consolaro et al. 2013). Several studies have demonstrated that free radicals can reach the surface beneath orthodontic brackets and adhesives on teeth and degrade the organic coloring molecules present throughout the tooth's labial surface, including beneath the bracket adhesive surface (Jadad et al. 2011; Marins et al. 2019; Silvestre et al. 2021).

However, the color variations between the nonbonded and bonded undersides of brackets when they are removed following whitening agent application during orthodontic treatment is controversial. Abas Frazão Marins et al. found that when whitening agents containing 35% HP were used directly on bonded orthodontic brackets, a consistent whitening effect was observed over the entire enamel surface, and no staining or shadows were observed after bracket removal. However, Sardarian et al. reported that 35% HP and 22% carbamide peroxide gel also whitened the area under the brackets; however, they may cause two‐colored appearance of teeth when the brackets are removed. de Melo Oliveira et al. reported that whitening agents containing 35% HP resulted in more heterogeneous tooth color owing to the presence of darker enamel around the periphery of the brackets (Sardarian et al. 2019; de Melo Oliveira et al. 2021).

The discoloration of dental materials is affected by several internal and external factors (Um and Ruyter 1991). Extrinsic factors of discoloration are known to cause staining of oral tissues and restorations, especially when combined with dietary factors (Um and Ruyter 1991; Guler et al. 2005). Previous studies have demonstrated that dental materials have low color stability owing to pigments found in foods and beverages (Faltermeier, Behr, and Müssig 2007b). Several studies have reported that staining beverages (coffee, coke, tea, and red wine) cause discoloration of dental materials (Faltermeier, Behr, and Müssig 2007b; Bahbishi et al. 2020; Alkhadim et al. 2020). Shin et al. observed color changes by immersing CAD/CAM blocks and three‐dimensional printed resins in food products with different coloring factors (grape juice, coffee, and curry). This study found that the discoloration of materials was caused by all foods, particularly curries that caused the most significant discoloration (Shin et al. 2020). Therefore, curry was selected as the staining solution in this study to provide a consistent and pronounced challenge to the resin brackets. This methodological choice was made to standardize the experimental conditions and minimize variability. Nonetheless, it is acknowledged that future studies should incorporate additional staining agents, such as black tea, coffee, and red wine, to better reflect diverse dietary habits and enhance the clinical applicability of the findings.

This study investigated the whitening effect of discolored resin brackets according to the concentration of HP and the number of treatment applications compared with the control group. Methods for measuring the whitening effect included visual observation using shade guides and quantitative evaluation using spectrophotometers, colorimeters, and imaging systems (Mounika et al. 2012; Chu et al. 2010; Kwon et al. 2015). Evaluation using shade guides is a common method in dentistry for clinical shade matching and is still considered a standard method in tooth whitening research (Chu et al. 2010; Paravina 2008, 2009). Therefore, this study used visual observations to record the color change of the resin bracket attached to the tooth surface before and after whitening treatment, to account for situations that are affected by the environment around the bracket. Our results demonstrated that all the experimental groups except groups HP 0, had a whitening effect with a significant color change of resin brackets following the third whitening application procedure. In the Group HP 8.7 and Group HP 17.5 groups, no significant difference was observed following the second whitening procedure compared to before the whitening agent application; however, a significant difference was observed after the third whitening application procedure. The present results do not correspond to those of previous studies that demonstrated a large color change after applying whitening agents at concentrations similar to those used in this study (Kurtulmus‐Yilmaz et al. 2013; Canay and Çehreli 2003). The discrepancy between these studies can be attributed to the application time of the whitening agents or the type of resin composites used. Hubbezoglu et al. reported that the HP 35 concentration had a significant whitening effect on the color change of the dental composite resins Admira and Durafill VS, which is consistent with the results of the present study (Hubbezoglu et al. 2008). In this study, Group HP 35 in particular demonstrated a significant whitening effect on the discolored bracket, with fewer treatments than the other groups. The greater whitening effect in Group HP 35 can be attributed to the application of whitening agents. This higher Group HP 35 concentration was expected to promote faster whitening of the resin bracket. Based on the above data, the null hypothesis stating that different HP concentrations have no effect on the color of the discolored resin brackets was rejected.

The SBS is one of the most important characteristics of brackets used for successful orthodontic treatment. It should be strong enough to withstand masticatory and orthodontic forces and easily removed without damaging the enamel during debonding (Miller et al. 2023; Azizi et al. 2020). During orthodontic treatment, the interface between the resin brackets and enamel can be loaded with maximum force. Therefore, SBS test was conducted to determine the bond strength (Beech and Jalaly 1980). Although the use of abrasive paper does not fully replicate clinical conditions, it was employed to minimize variability caused by differences in the natural enamel surface. This standardization process was essential for controlling potential confounding variables and ensuring the reliability and reproducibility of the SBS measurements. In this study, similar values were found between the experimental and control groups when examining the SBS in the teeth. This means that no significant difference was observed in the SBS between the two groups. These values were higher than the clinically acceptable values of 6–8 MPa (Reynolds 1975). Bishara et al. applied a 25% HP gel to the teeth and exposed them to a light source for 20 min; this procedure was performed twice. The brackets were subsequently bonded and the bond strength was measured; no decrease in the bond strength was observed (Bishara et al. 2005). This finding is consistent with the results of the present study. In addition, Sterrett and Haywood et al. reported that no alterations were observed except for normal morphological variations in the enamel surface at 3%–35% HP solutions (Sterrett et al. 1995; Haywood et al. 1990). Therefore, the null hypothesis that different HP concentrations do not affect the SBS between discolored resin brackets and enamel was accepted.

In contrast, a previous study in which a 35% HP gel was applied three times at 1‐week intervals demonstrated a decrease in the bond strength when compared to the non‐whitening control group (Sardarian et al. 2019). Oltu and Gurgan found that whitening agents containing low concentrations of HP did not affect the enamel structure, whereas those containing high concentrations of HP did (Oltu and Gürgan 2000). Therefore, we suggest that a whitening treatment based on a concentration in the range of 8%–10% does not affect the bond strength and whitening effect on the bracket (Jadad et al. 2011; Montenegro‐Arana et al. 2016).

The ARI score is clinically important because the lower the bond strength at the interface of the enamel and orthodontic adhesive, the greater would be the stress applied to the enamel surface (Oztas et al. 2012). According to previous studies, higher ARI scores indicate high bonding strength of the bracket and adhesive interface and lower risk of enamel damage. Sardarian et al. demonstrated no statistically significant difference in the ARI scores between the 35% HP and 22% carbamide peroxide groups (*p* = 0.38) (Sardarian et al. 2019). Akin et al. also found no significant difference in the ARI scores between the 38% HP and 10% carbamide peroxide groups (*p* > 0.05) (Akin et al. 2013). In the two previous studies, the frequency of score 0, where no adhesive remained on the enamel surface, was mostly high; however, the results of this study demonstrated that all the groups had a greater frequency of score 1, which implies that less than 50% of the adhesive remained on the enamel surface, or score 3, which implies that all the adhesive remained on the tooth with distinct impression of the bracket base. In particular, Group HP 17.5 had a greater frequency of scores of 3, which indicates less damage to the enamel on subsequent removal (Sardarian et al. 2019; Akin et al. 2013).

The hardness of the resin bracket affects the capacity of the appliance to withstand loads and maintain surface structural integrity in the presence of loads arising from mechanics, such as arch wire sliding, formation of high torque moments, or masticatory forces generated when chewing hard foods. The synergistic action of several factors, such as cyclic loading, temperature fluctuations, and acidic environments, may result in a reduced fatigue limit for plastic brackets, with effects induced by a variety of deformations, including shear yielding, disentanglement, slip of chain segments, and cracking, as opposed to dislocation sliding along the crystallographic planes predominant in metals (ZINELIS et al. 2005).

Previous studies have measured the microhardness of enamel after whitening, but none have measured the microhardness of brackets themselves following whitening procedure during orthodontic treatment (Al‐Angari et al. 2021; Magalhães et al. 2012; Pujari et al. 2016). Therefore, comparison with previous studies is limited. Thus, we compared our findings with those of previous studies on composite resins using comparable parameters. Saeed et al. reported that the microhardness of flowable composite resins and compomers significantly decreased following application of 6% carbamide peroxide and increased in packable composite resins, indicating that whitening agents have a significant effect on the hardness depending on the material (Saeed et al. 2021). Fernandes et al. and Polydorou et al. reported that the microhardness values of composite resins whitened with carbamide peroxide and HP were maintained (Fernandes et al. 2020; Polydorou et al. 2007). These differences can be explained by differences in the type of composite resin, monomer composition, degree of polymerization, filler size, particle distribution, filler content, type of whitening agent, and protocol (Fernandes et al. 2020). When comparing the results of this study with those of previous studies on composite resins, differences in the results would probably occur owing to the differences in the measurement parameter, material composition, type of whitening agent, and whitening protocol.

In this study, the microhardness of the bracket surface according to the concentration of HP was significantly different between HP 0 and HP 35, and no statistically significant difference was observed between the other groups. Therefore, the null hypothesis that the HP concentration does not affect the microhardness of the bracket surface was rejected. The mean and standard deviations of the microhardness of each group were 13.23 ± 0.69 (HP 0), 12.91 ± 0.21 (HP 8.7), 12.93 ± 0.17 (HP 17.5), and 12.69 ± 0.17 (HP 35), and the raw material of the resin bracket used in this study was polycarbonate. Zinelis et al. reported the microhardness of a rod‐ and disk‐shaped polycarbonate specimen to be 14.92 ± 0.82 and 14.21 ± 1.02, respectively (Zinelis et al. 2005). Based on the above results, HP reduced the microhardness of the resin brackets; however, the effect was insignificant. Nevertheless, since the results were statistically significant in 35% of the cases, HP concentrations between 8% and 17.5%, which have proven whitening effects in this study, are recommended.

The SEM analysis provided information on the surface characterization and topography of the test object. Therefore, a change in the surface characteristics of the resin bracket following whitening treatment can be seen in the SEM images. The SEM images of the specimens in the experimental and control groups did not show any noticeable differences. Therefore, the null hypothesis that the concentration of HP during the whitening treatment would not affect the surface microstructure of the discolored resin brackets was accepted.

## 5 Limitations and Future Directions

Because the specimens were stored in distilled water after applying HP to the discolored resin brackets in this study, it was not possible to reproduce the effect of remineralization by saliva and the restoration of porosity on the surface to alleviate the surface changes. Therefore, deviations may have occurred in vivo. In addition, the material used in this study contained only HP, the primary active ingredient, unlike commercial whitening products that often include pH adjusters and wetting agents to enhance their efficacy and stability. As a result, variations in the effects of whitening treatments may exist, particularly when compared to products designed for clinical use. The application protocol of HP in this study was designed to simulate in‐office teeth whitening protocols commonly employed in clinical settings. This approach ensured consistent and controlled experimental conditions, allowing for reliable evaluation of HP's whitening efficacy on resin brackets. However, it is acknowledged that differences may exist between the experimental conditions and practical applications in clinical scenarios. Moreover, a study reported that the whitening results were more effective when light was irradiated with a whitening agent containing 25% HP; thus, further research on the whitening effect of resin brackets with or without light irradiation for each concentration is warranted (Ontiveros and Paravina 2009). Generalizing the results of this study is challenging because only one type of resin bracket and discoloring method was employed. Additionally, although color assessments were conducted under standardized conditions by a single trained investigator, intra‐ and interrater reliability were not quantitatively assessed using intraclass correlation coefficients (ICC). Future studies should explicitly incorporate ICC analyses with repeated evaluations to validate and enhance the reliability of color measurements.

Despite the above limitations, when whitening was performed on the resin brackets, a concentration of 8.7% and 17.5% HP produced effective whitening without significantly affecting the properties of the brackets and teeth. However, selective whitening of the resin brackets, excluding the enamel, should be recommended. It is important to note that HP‐based whitening procedures are not without risks. Previous studies have reported potential adverse effects, including increased tooth sensitivity, alterations to enamel surfaces, and gingival irritation, particularly when high concentrations or prolonged application times are used (Tay et al. 2009). These considerations underscore the necessity of controlled application and proper clinical supervision to ensure the safety and efficacy of whitening treatments for resin brackets. Additionally, some studies have suggested that whitening procedures performed with brackets bonded to the tooth are more likely to result in surface staining on enamel following bracket removal (Consolaro et al. 2013). This highlights the importance of developing optimized whitening protocols that minimize such risks while maintaining esthetic outcomes.

## 6 Conclusion

Based on the results, the application of HP at concentrations of ≥ 8.7% and < 17.5% effectively whitened discolored resin brackets without significantly affecting their bond strength, microhardness, or surface microstructure. These findings highlight the potential of HP as a viable whitening treatment for orthodontic patients with resin brackets, addressing a common esthetic challenge caused by discoloration from dietary pigments.

## Author Contributions

**Song‐Yi Yang:** conceptualization, methodology, investigation, supervision, project administration, and reviewing the paper.

## Conflicts of Interest

The author declares no conflicts of interest.

## Data Availability

The data that support the findings of this study are available from the corresponding author upon reasonable request.
